# Exploring the Role of GDF-15 in Inflammatory Bowel Disease: A Case-Controlled Study Comparing Crohn’s Disease and Ulcerative Colitis with Non-Inflammatory Controls

**DOI:** 10.3390/metabo14040185

**Published:** 2024-03-25

**Authors:** Ondřej Kučerka, Marie Blahutová, Vít Kosek, Petra Mináriková, Jan M. Horáček, Petr Urbánek, Martin Malý

**Affiliations:** 1 Department of Medicine, First Faculty of Medicine, Charles University and Military University Hospital Prague, 169 02 Prague, Czech Republic; ondrej.kucerka@uvn.cz (O.K.); petra.minarikova@uvn.cz (P.M.); petr.urbanek@uvn.cz (P.U.); 2 Department of Military Internal Medicine and Military Hygiene, Military Faculty of Medicine, University of Defence, 500 02 Hradec Kralove, Czech Republic; jan.horacek@unob.cz; 3 Department of Clinical Biochemistry, Military University Hospital, 169 02 Prague, Czech Republic; marie.blahutova@uvn.cz; 4 Department of Food Chemistry and Analysis, University of Chemistry and Technology, 160 00 Prague, Czech Republic; vit.kosek@vscht.cz

**Keywords:** GDF-15, Inflammatory Bowel Disease, Crohn’s disease, ulcerative colitis

## Abstract

Inflammatory bowel disease, encompassing Crohn’s disease and ulcerative colitis, is a persistent immune-mediated inflammatory gastrointestinal disease. This study investigates the role of growth differentiation factor 15 in severe IBD cases, aiming to identify a reliable parameter to assess disease severity and monitor activity. We analyzed plasma samples from 100 patients undergoing biologic therapy for severe IBD and 50 control subjects. Our analysis included evaluations of GDF-15 levels, inflammatory markers, and clinical features. We employed statistical methods such as the Mann–Whitney U test, ANOVA, and Spearman’s correlation for an in-depth analysis. Our results demonstrated consistently higher GDF-15 levels in patients with both Crohn’s disease and ulcerative colitis compared to the control group, irrespective of the biologic treatment received. The correlation analysis indicated significant relationships between GDF-15 levels, patient age, fibrinogen, and IL-6 levels. This study positions GDF-15 as a promising biomarker for severe IBD, with notable correlations with age and inflammatory markers. These findings underscore GDF-15’s potential in enhancing disease monitoring and management strategies in an IBD context and encourage further research to clarify GDF-15’s role in the IBD pathophysiology.

## 1. Introduction

Inflammatory bowel disease (IBD) is a chronic condition characterized by inflammation of the gastrointestinal tract, often accompanied by systemic inflammation and extraintestinal manifestations. It encompasses Crohn’s disease (CD) and ulcerative colitis (UC), both of which are chronic, incurable diseases with a pathogenesis that remains not fully understood. The development of these diseases involves an intricate interplay of genetic and environmental factors, which disrupt the immune system’s function. IBD commonly affects adolescents and adults, with an equal incidence in males and females. Both diseases share similar symptomatology, including abdominal pain, diarrhea, weight loss, and rectal bleeding.

Growth differentiation factor 15 (GDF-15), identified in 1997 by Bootcov et al. [1], is known by various names, including placental transforming growth factor-β (TGF-β) [2], placental bone morphogenetic protein [3], macrophage inhibitory cytokine-1 [1], and non-steroidal anti-inflammatory drug-activated gene-1 [4]. It is a peptide hormone belonging to the TGF-β superfamily [1,2] that plays critical roles in embryonic development and cellular processes such as homeostasis, cell growth, differentiation, proliferation, migration, adhesion, and apoptosis [5,6,7,8,9]. TGF-β superfamily proteins are expressed as precursors, stored in the extracellular matrix, and activated in response to stimuli like inflammation [10,11].

The GDF-15 gene is located on chromosome 19p12.1–13.1 and circulates as a 25 kDa homodimer composed of two 112 amino acid chains [12]. Under normal conditions, GDF-15 is minimally expressed in various tissues, including the lung, mammary gland, liver, pancreas, kidney, peripheral and central nervous systems, and gastrointestinal tract [13,14,15,16]. Elevated levels are observed in the placenta and prostate [17,18]. Functionally, GDF-15 acts as a hormone and a stress-induced cytokine or stress-sensitive factor [19,20], with a typical serum concentration of around 450 pg/mL [21].

Elevated GDF-15 levels have been observed in other inflammatory conditions, such as rheumatoid arthritis [22,23,24], psoriasis [25], and Behçet’s disease [26], with some studies demonstrating a correlation between serum GDF-15 levels and disease activity [23,26,27]. This study specifically aims to evaluate serum GDF-15 levels in patients with severe forms of CD and UC.

## 2. Materials and Methods

### 2.1. Study Design

In this study, we conducted a comparative analysis of plasma samples from two distinct groups: an IBD group and a control group. The IBD group comprised 100 patients diagnosed with severe CD or UC, all of whom were undergoing biologic therapy. The control group consisted of 50 individuals without any chronic inflammatory disease. To ensure the integrity of the control group, we established specific exclusion criteria, including the presence of any acute illness, pregnancy, and a history of chronic inflammatory disease, malignancy, or prostate disease. Both groups were recruited from the Military University Hospital in Prague, Czech Republic.

The primary aim of the analysis was to assess and compare the plasma levels of GDF-15 in both groups. Additionally, we sought to investigate the correlation between GDF-15 levels and various inflammatory markers, including leukocytes, high-sensitivity C-reactive protein (hsCRP), interleukin-6 (IL-6), and fibrinogen.

### 2.2. Sample Collection and Preparation

Blood samples for the study were collected through venipuncture during scheduled follow-up visits at the IBD Biological Treatment Center, Military University Hospital Prague, Czech Republic, between March 2022 and June 2023. Patients from both the IBD and control groups had their blood samples drawn into vacuum tubes containing EDTA. These samples were then immediately centrifuged at 3300× *g* for 15 min at 12 °C to obtain plasma, which was aliquoted into eight tubes per subject and stored in the dark at −80 °C until the time of analysis.

On the day of analysis, a single aliquot from each subject was thawed and brought to room temperature. After thorough mixing, the prepared samples were pipetted into labeled sample cups for analysis using a COBAS PRO analyzer (Roche, Basel, Switzerland). The results were obtained from the calibration curve, which included two-point calibration performed for the instrument, alongside the master curve provided by the device manufacturer. The total determination time for each sample was approximately 18 min, with the lowest detectable level of the assay being 400 ng/L.

### 2.3. Statistical Analysis

The Mann–Whitney U-test, Kruskal–Wallis test, χ^2^ test, and correlation and associated analyses, such as the Shapiro–Wilk test, were performed in JASP 0.18.1.0 (University of Amsterdam). The receiver operating characteristic area under the curve (ROC-AUC) was calculated in R, using the package ROCit in RStudio 2023.12.1 Build 402 with R version 4.0.2.

## 3. Results

### 3.1. Demographic and Clinical Characteristics

**Figure 1:**
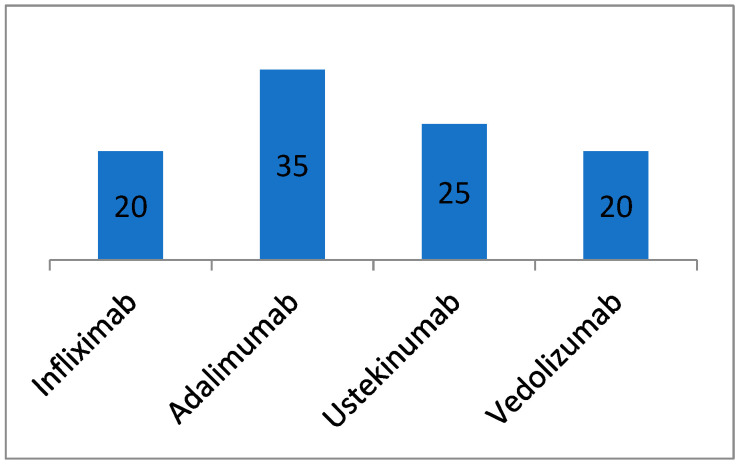
Number of patients by type of biological treatment.

In this study, we enrolled 100 patients with IBD and compared their clinical parameters with those of 50 control subjects. The IBD group comprised 77 patients with severe CD and 23 patients with severe UC. All patients in the IBD group were undergoing biologic therapy, as depicted in Figure 1. Among the IBD cohort, there were 60 males (60%) and 40 females (40%), with a mean age of 43 years (standard deviation ± 9). Within this group, 18 subjects (18%) were identified as smokers. The control group included 21 males (42%) and 29 females (58%), with a similar mean age of 43 years (standard deviation ± 10); this group had four smokers (8%). A chi-squared analysis indicated a significant difference in gender distribution between the groups (*p* = 0.037), while differences in smoking status were not significant (χ^2^ = 2.66, *p* = 0.103). Furthermore, the Mann–Whitney U test revealed no significant age difference between the two groups (W = 2554.5, *p* = 0.829), as shown in Table 1, Table 2 and Table 3.

### 3.2. GDF-15

**Figure 2:**
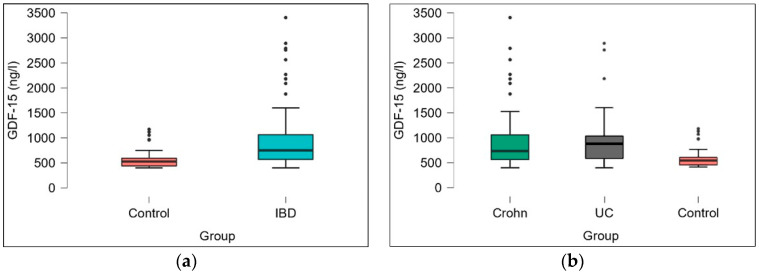
(**a**) GDF-15 levels in the IBD group and the non-inflammatory control group; (**b**) GDF-15 levels in patients with Crohn’s disease (Crohn), patients with ulcerative colitis (UC), and patients without inflammatory diseases (control).

Our study found that the plasma GDF-15 levels were significantly higher in patients with IBD compared to the control group, as determined by the Mann–Whitney U test (*p* < 0.001). Further analysis using ANOVA to separately compare the Crohn’s disease group, the ulcerative colitis group, and the control group revealed consistently elevated plasma GDF-15 levels in both CD and UC (Kruskal–Wallis test, *p* < 0.001), as illustrated in Figure 2. Notably, the type of biologic treatment did not influence the GDF-15 levels.

In our correlation analysis using Spearman’s correlation test, we observed that increased GDF-15 levels were associated with age (ρ = 0.398, *p* < 0.001), fibrinogen (ρ = 0.363, *p* < 0.001), and IL-6 levels (ρ = 0.299, *p* < 0.001). However, the association of GDF-15 with hsCRP was relatively weak (ρ = 0.256, *p* = 0.002). An evaluation of GDF-15 as a marker to discriminate IBD patients from controls was also performed using ROC analysis, with a calculated AUC of 0.7473, indicating acceptable performance.

### 3.3. Conventional Markers of Inflammation

Of the serum biomarkers, the determination of CRP is the gold standard in the management of IBD patients with both symptomatic and asymptomatic disease [22]. We therefore correlated GDF-15 with CRP and other inflammatory parameters routinely measured in our hospital, such as leukocytes, fibrinogen, and IL-6. These conventional markers of inflammation were determined simultaneously with GDF-15 in both groups of patients. In the IBD group, all inflammatory markers were significantly higher than in the control group, as indicated in Table 4 and Table 5. This was despite the fact that all IBD patients were in remission at the time of blood sampling. Clinical remission is defined as the resolution of abdominal pain and the resolution of altered bowel habits [23].

## 4. Discussion

GDF-15, a multifunctional protein with diverse roles in health and disease, is expressed in numerous human tissues and is responsive to cellular stress. It has emerged as a significant player in various diseases, owing to its ready detectability in human plasma, which has led to its extensive utilization as a biomarker in countless studies. The associations of GDF-15 extend across a spectrum of conditions, including diabetes mellitus [24,25], cancer [26], cachexia [27], chronic inflammatory diseases [28,29,30], cognitive impairment [31,32], heart failure [33], coronary artery disease [34,35,36,37], and atrial fibrillation [38]. Its dysregulation is implicated in various pathological conditions, making it a potential therapeutic target and diagnostic biomarker for several diseases.

The exact pathophysiological effects of GDF-15 are still under intense investigation. Regarding cardiovascular effects, GDF-15 is thought to be involved in the inhibition of myocardial hypertrophy and may exert both atherogenic and antiatherogenic effects [39,40,41,42]. In the kidney, it exhibits a protective and antifibrotic effect [43,44], while, in the brain stem, it induces appetite suppression through the activation of the GFRAL receptor in the area postrema [45,46,47]. However, in the context of tumors, the data on the effect of GDF-15 are controversial, with some authors suggesting its carcinogenic activity and others pointing to its tumor suppressor activity [14,48,49,50]. Wallin et al. analyzed the expression of GDF-15 in the tumor tissue of patients with colorectal cancer. They showed that a high level of GDF-15 expression in the tumor tissue and high levels of GDF-15 in the plasma correlated with an increased risk of recurrence and reduced overall survival [51]. The study conducted by Vocka et al. [52] revealed the promising potential of GDF-15 as a biomarker in patients with metastatic colorectal cancer. Their findings demonstrated that GDF-15 exhibited comparable sensitivity to carcinoembryonic antigen (CEA) while also displaying a noteworthy correlation with liver involvement, in contrast to CEA. To the best of our knowledge, there have been no studies investigating the marker GDF-15 in non-neoplastic intestinal disorders.

Our finding aligns with several studies in rheumatoid arthritis (RA) patients, psoriasis patients, or Behçet’s disease (BD) patients, where elevated GDF-15 levels were reported [28,29,30,53,54,55,56]. Esalatmanesh et al. [53] suggested that GDF-15 levels might correlate with RA activity. Similarly, He et al. [28] observed that RA patients with positive CRP had higher plasma GDF-15 expression than those with negative CRP. In our IBD cohort, we noted a weak correlation between GDF-15 levels and both CRP and hsCRP (the hsCRP test is a highly sensitive quantification of CRP). Since CRP is a widely recognized marker of inflammation, its elevation in CD and UC typically indicates active inflammation. However, the variability in correlation strength among individuals suggests that GDF-15 might be a more reliable indicator of disease severity, activity, or the body’s response to disease.

Tasolar et al. [55] investigated GDF-15 in patients with psoriasis. The study included 50 psoriasis patients and 32 controls. The psoriasis patients were divided into three groups based on the Psoriasis Area Severity Index score (PASI, a tool used to measure the severity and extent of psoriasis): patients with mild psoriasis (PASI < 10), patients with moderate psoriasis (PASI 10–20), and patients with severe psoriasis (PASI > 20). No or statistically insignificant differences were observed between psoriasis patients and healthy controls for interleukin-12 (IL-12), interleukin-17a (IL-17a), interleukin-22 (IL-22), and interleukin-23 (IL-23). In contrast, a statistically significant difference was observed for hsCRP, tumor necrosis factor-alpha (TNF-α), and GDF-15. GDF-15 was significantly higher in all three patient groups (mild, moderate, severe psoriasis). Furthermore, this study confirmed the strong correlation between the disease duration and PASI score. A serum GDF-15 level above 1498 pg/mL was identified as potentially predictive of a high PASI score. Akbari et al.’s case–control study compared 45 psoriatic patients with 45 healthy individuals. They demonstrated a significant association between serum GDF-15 levels and psoriasis, as well as between GDF-15 gene expression and psoriasis. Both serum GDF-15 levels and GDF-15 gene expression correlated with disease severity [29].

Elbarky et al. [30] compared serum GDF-15 levels in 30 patients with BD and 20 controls. Sarıyıldız et al. [56] compared serum GDF-15 levels in 46 patients with BD and 30 healthy subjects. In both studies, the BD patients were divided into two groups according to the presence of peripheral arthritis and arthralgia. In both studies, the authors reached similar conclusions, showing that BD patients with peripheral arthritis and arthralgia had significantly higher serum GDF-15 levels than patients without peripheral arthritis or controls. These findings may be explained by the increased GDF-15 expression in activated macrophages after stimulation with proinflammatory cytokines such as TNF-α, interleukin-1β (IL-1β), and IL-6 in the synovium during inflammation [40]. Therefore, GDF-15 could be used as a marker for peripheral arthritis in BD patients.

A team of authors from the University of Colorado demonstrated significantly elevated plasma levels of GDF-15 in patients with systemic sclerosis-associated pulmonary arterial hypertension (SSc-PAH) compared to SSc patients without PAH. The GDF-15 levels were positively correlated with echocardiographic estimates of right ventricular systolic pressure and plasma levels of the amino-terminal propeptide form of brain natriuretic peptide (NT-proBNP). Plasma GDF-15 levels greater than 125 pg/mL were associated with reduced survival. Additionally, the expression of the GDF-15 protein was found to be increased in the lung tissue of patients with SSc-PAH [57].

CRP is the most commonly used serum biomarker of inflammation in IBD patients. However, CRP is not disease-specific [58]. In patients with CD who are in symptomatic remission, CRP levels below 5 mg/L indicate the absence of active inflammation [22]. In their investigation, Con et al. [59] examined the dynamics of C-reactive protein (CRP) in individuals with acute severe ulcerative colitis (UC) following the administration of infliximab as a salvage therapy. This comprehensive study included a cohort of 94 patients, 20% of whom required colectomy within 12 months. At the end of the study, patients were stratified into two distinct categories—those who did not require colectomy and those who did. Notably, the baseline CRP levels showed no statistically significant difference between the two cohorts (colectomy group: median 65 mg/L, non-colectomy group: median 46 mg/L). On the first day after infliximab administration, CRP levels showed a significant decrease in both groups (colectomy group: median 32 mg/L, non-colectomy group: median 11 mg/L). On the third day after infliximab administration, the median CRP levels continued to decrease to 17 mg/L and 5 mg/L, respectively.

Considering these findings, the monitoring of GDF-15 levels alongside traditional markers like CRP, complete blood counts, or fecal calprotectin could offer a more comprehensive assessment of the inflammatory status in IBD patients, thereby enhancing the disease management strategies. Future longitudinal studies should focus on varying treatment modalities, such as aminosalicylates, corticosteroids, and immunosuppressants, to understand how GDF-15 levels evolve with different therapeutic interventions. This approach could reveal patterns linked to the treatment response and disease progression. Comparisons of the GDF-15 levels between biologic-naive patients and those on various biologic regimens could elucidate the biologics’ specific impact on GDF-15 and its potential as a marker for the treatment response.

By monitoring the GDF-15 levels at strategic time points, we could assess its value as a predictive marker for disease flare-ups or complications in IBD patients. This would have significant implications in terms of tailoring individual treatment plans and facilitating early intervention, ultimately improving patient outcomes.

Overall, while CRP is a valuable biomarker in IBD management, GDF-15 may potentially offer certain advantages in terms of specificity, early detection, correlations with disease activity, and predictive value. However, further research is needed to fully validate its utility and establish its clinical significance in routine practice.

## 5. Study Limitations

- This was a small, single-center, cross-sectional study designed to compare the plasma GDF-15 levels between patients with CD and UC receiving biologic therapy and a control group.
- The study primarily focused on patients with severe IBD undergoing biologic therapy, which may limit the generalizability of the findings to patients with less severe forms of IBD or those not receiving biologic treatment.
- While the study identified associations between plasma GDF-15 levels and severe IBD, the clinical significance of these findings in terms of diagnosis, prognosis, or treatment remains to be fully elucidated.

## 6. Conclusions

- Patients with severe IBD, including CD and UC, undergoing biologic therapy, exhibited significantly higher levels of GDF-15 in their plasma compared to individuals without chronic inflammatory diseases.
- There was a positive correlation between elevated GDF-15 levels and other markers of inflammation, such as age, fibrinogen, and IL-6, indicating that GDF-15 may serve as a complementary marker in assessing the disease status in IBD patients.
- Further research is warranted to uncover the specific mechanisms underlying GDF-15 dysregulation in IBD and to explore its potential as a biomarker for disease activity and the treatment response.

## Figures and Tables

**Table 1:** Patient characteristics.

Characteristics	IBD Patients	Controls	*p*-Value	Test
	*n* = 100	*n* = 50		
Age (y)	43 (25–67)	43 (18–61)	0.829	U-test
Sex (male)	60 (60%)	21 (42%)	0.037	χ^2^
Sex (female)	40 (40%)	29 (58%)	0.037	χ^2^
Current smoker	18 (18%)	4 (8%)	0.103	χ^2^
Body mass index	25			
Total protein (g/L)	73.6	71	0.003	*t*-test
Albumin (g/L)	44.2	44.6	0.449	*t*-test
Abdominal surgery	71 (71%)			
Disease duration (y)	13.4			

**Table 2:** Number of patients by disease phenotype.

CD	*n* = 77
inflammatory	38
stricturing	10
fistulizing	29
**UC**	***n* = 23**
extensive colitis	17
left-sided colitis	5

**Table 3:** Treatment of IBD patients and their chronic inflammatory comorbidities.

Type of Treatment	Number of Patients
biologic therapy	100 (100%)
5-aminosalicylic acid (5-ASA)	63 (63%)
azathioprine	8 (8%)
methotrexate	1 (1%)
corticoids	2 (2%)
**Concomitant chronic inflammatory diseases**	**Number of patients**
autoimmune thyroiditis	2
celiac disease	1
psoriasis	5
hidradenitis suppurativa	2
atopic eczema	2
antiphospholipid syndrome	1

**Table 4:** GDF-15 and inflammatory markers.

		Median	Mean	Std. Deviation	Minimum	Maximum
GDF-15 (ng/L)	Control	527	569.34	188.655	400	1171
	IBD	751	943.23	608.579	400	3406
Leukocytes (×10^9^/L)	Control	6.095	6.338	1.703	3.44	11.58
	IBD	6.84	7.109	1.876	3	13.17
hsCRP (µg/L)	Control	817.55	2113.322	2611.358	25	9461
	IBD	1511.7	2977.522	3811.365	87.6	24,497
IL-6 (ng/L)	Control	1.5	1.776	0.617	1.5	4.3
	IBD	1.6	2.751	2.556	1.5	18
Fibrinogen (g/L)	Control	2.62	2.668	0.483	1.77	4.04
	IBD	2.82	2.861	0.474	1.8	4.11

**Table 5:** Inflammatory markers.

Mann-Whitney U Test.		
	W	*p*
GDF-15	1263.5	<0.001
Leukocytes	1867.5	0.012
hsCRP	1970	0.035
IL-6	1825.5	0.003
Fibrinogen	1902.5	0.017

## Data Availability

The data presented in this study are available on request from the corresponding author. The data are not publicly available because they contain information that could compromise the privacy of the research participants.
